# A river, frontline families, and the double-edged sword of community participation: how nutrition interventions are shaped at the village level in Dhubri, India

**DOI:** 10.1093/heapol/czag011

**Published:** 2026-06-29

**Authors:** J R Jith, S Dash, R Bedamatta

**Affiliations:** The George Institute for Global Health India, 308, Third Floor, Elegance Tower, Plot No. 8, Jasola District Centre, New Delhi 110025, India; The George Institute for Global Health India, 308, Third Floor, Elegance Tower, Plot No. 8, Jasola District Centre, New Delhi 110025, India; Department of Humanities and Social Sciences, Indian Institute of Technology Guwahati, Guwahati 781039, Assam, India

**Keywords:** nutrition interventions, community participation, village-level health systems, frontline health workers, frontline families, Dhubri

## Abstract

This paper explores how top-down health interventions engage with and reshape communities at the village level, focusing on nutrition interventions as tracers to examine the functioning of local health systems. Drawing on 28 in-depth interviews and 6 focus group discussions conducted in Dhubri district, Assam, the study investigates how such interventions are delivered through a health system constituted of both formal structures and informal arrangements, deeply influenced by the ecology of the Brahmaputra River. Using an exploratory qualitative approach, the study surfaces five key themes. First, seasonal disruptions linked to the river significantly affect service continuity, revealing the inadequacy of existing policies to respond to environmental vulnerability. Second, the creation of ‘frontline families’—where the personal lives and unpaid labour of frontline health workers’ households are drawn into service delivery—reflects systemic dependence on invisible support. Third, community participation emerges as a double-edged sword, simultaneously enabling programme reach and generating unrealistic demands and burdens on frontline actors. Fourth, informal spaces and interpersonal relationships underpin much of the operational success of interventions, compensating for gaps in formal governance and infrastructure. Finally, symbolic campaigns, detached from local material realities, often foster alienation rather than awareness. The findings call for a rethinking of intervention design and delivery that take account of embedded social, ecological, and institutional realities. The paper argues that building just and sustainable health systems requires recognising and supporting the informal, relational, and contextual dimensions that currently remain overlooked in dominant policy frameworks.

Key messages

- The Brahmaputra River and its seasonal rhythms profoundly shape the delivery and continuity of nutrition interventions in Dhubri, exposing a critical gap in policy design which assumes environmental stability.
- Frontline health workers in such settings rely heavily on informal support systems, including their own family members, to meet excessive demands, revealing a hidden layer of unpaid labour that sustains public health delivery at great personal cost.
- Community participation, though central to current health policy discourse, can place disproportionate expectations on frontline workers and sometimes reinforce inequities, unless accompanied by structural support and clarity of roles.
- Informal spaces and interpersonal relationships—not formally recognised in policy—function as critical infrastructure for health-service delivery, underscoring the importance of acknowledging and strengthening these relational and adaptive capacities within health systems.

## Introduction

Malnutrition remains a critical global health challenge, with India bearing a significant burden of the world’s undernourished children. The country accounts for >31% of the global burden of stunted children, nearly 18% of global wasting cases, and has one of the highest absolute numbers of children under the age of 5 years affected by undernutrition globally. In 2022, an estimated 36.1 million children in India under the age of 5 years were stunted, and 13.3 million were wasted, including 3.2 million severely wasted (UNICEF et al. 2023). Despite large-scale, ongoing national-level evidence-based nutrition interventions, such as the Integrated Child Development Services (ICDS) scheme, operational since 1975, and the more recent National Health Mission (NHM) with its community-based nutrition programmes, high prevalence rates persist. This is particularly evident in economically disadvantaged regions like Assam, with marked district-level disparities (IIPS & ICF 2021, NITI Aayog 2021).

Addressing undernutrition effectively requires recognising it as a multifaceted problem influenced by broader social determinants such as poverty, education, gender inequality, and inadequate health infrastructure (Bhutta et al. 2013, Black et al. 2013). Additionally, local contexts significantly shape the implementation and effectiveness of nutrition-specific interventions, particularly at the grassroots levels where health services interact directly with communities (Menon et al. 2018, Kim et al. 2019). Understanding how health systems operate at the village level and how they mediate between policy frameworks and local realities is crucial yet under-explored within public health nutrition research.

The need for this particular study emerged following a survey on the nutritional status of children in Assam conducted during 2021–2022, revealing significant inter-district variations in anthropometric failure and its determinants, including the coverage of national nutrition interventions. This survey identified Dhubri district as starkly lagging in these indicators. While the survey highlighted the ‘what’ and ‘where’ of these disparities, it underscored the need to understand the ‘how’ and ‘why’ at the local level. Recent scholarship increasingly emphasises the need to explore health-system interventions through the lens of social context and everyday practice to achieve sustainable, contextually sensitive outcomes (de Savigny and Adam 2009, George et al. 2018). Qualitative methods, with their capacity to capture complexities, social dynamics, and institutional interactions, offer powerful tools for exploring these micro-level health system characteristics (Gilson et al. 2011, Sheikh et al. 2011).

In addition to its poor nutrition indicators, Dhubri represents a region of systemic invisibility—geographically peripheral, ecologically fragile, and politically marginalized. Historically viewed as a ‘remote border district’, its communities have long experienced governance neglect, limited infrastructural investment, and persistent administrative underreach (Nath et al. 2021, Deka and Kalita 2024, Saikia and Mahanta 2024).

This makes Dhubri a critical site for examining how health interventions unfold in spaces of spatial and social exclusion. Understanding Dhubri’s health system requires situating the district within its wider historical and political ecology. A defining geographic feature of Dhubri is the extensive presence of Char-Chapori (riverine island and riverbank) settlements, which are formed by the deposition of silt from the Brahmaputra. These are dynamic landforms with high soil fertility and extreme ecological vulnerability, experiencing constant cycles of flooding, erosion, and reformation.

Many residents of the Char-Chapori areas are descendants of peasants from East Bengal who were encouraged by British colonial administrators in the late nineteenth and early twentieth centuries to settle and cultivate Assam’s floodplains, then described as ‘fallow wastelands’ of the recently annexed province. Zamindars in Assam, aligned with the colonial state’s revenue-maximizing ambitions, also welcomed these settlers to cultivate Char-Chapori areas, and jute soon emerged as the principal cash crop. By the early twentieth century, the Brahmaputra’s floodplains had become the Empire’s easternmost jute frontier (Saikia 2015, Kumar and Das 2019, Saikia 2019).

In the post-colonial period, particularly after the 1971 Bangladesh Liberation War, these same communities became central to Assam’s anxieties around migration and citizenship. The Assam Anti-Foreigners Agitation (1979–1985) and later the National Register of Citizens exercises produced new forms of administrative precarity, characterized by insecure land tenure, limited documentation, and restricted access to welfare entitlements (Guha 2019, Hazarika 2000, Sultana 2024).

The accelerating impacts of climate change, manifested in erratic rainfall, prolonged flood cycles, and shifting sedimentation, further strain this fragile infrastructure (Bhuyan et al. 2025, Saikia and Mahanta 2025). The district experiences among the highest rates of riverbank erosion and population displacement in Assam. Recurrent environmental shocks undermine livelihoods, disrupt food security, and have a direct impact on health service delivery. Health facilities are periodically rendered inaccessible or destroyed, community health workers face acute mobility constraints, and displaced families often lose entitlements tied to fixed locations or official documentation (Dekaraja and Mahanta 2022).

This qualitative study envisions bridging the existing research gap by exploring how village-level health systems function in Dhubri, particularly regarding the implementation of nutrition interventions. It addresses two core research questions. (i) What are the actors and institutions that constitute the village-level health systems in Dhubri district? (ii) How does the social construct of these health systems influence the delivery and effectiveness of nutrition-specific interventions? By addressing these questions, this paper seeks to contribute valuable insights to health policy and systems research, particularly in formulating context-sensitive, effective, and equitable nutrition interventions.

This study also responds to calls for a granular, context-sensitive inquiry into how health systems function as socially embedded constructs (Gilson et al. 2011, Sheikh et al. 2011). Applying concepts such as systems thinking, actor–network perspectives, and embedded governance (de Savigny and Adam 2009, George et al. 2018), we investigate the dynamics of village-level health systems in Dhubri through the lens of nutrition interventions, treating them as ‘tracers’ that help uncover how policies are translated, negotiated, and adapted within everyday practices of frontline health delivery.

This paper begins with an overview of the study setting, outlining its ecological vulnerabilities, infrastructural challenges, and socio-political marginalization. We then detail the data collection and analysis methods. The findings are presented thematically, exploring how nutrition interventions are shaped by Dhubri’s riverine geography, seasonal disruptions, governance practices, community expectations and interactions, and the lived realities of frontline workers. The discussion reflects on the implications of these findings for research, policy, and practice, and concludes with recommendations for strengthening the delivery of nutrition interventions in similar contexts and for broader health-system strengthening.

## Methods

### Study setting

Dhubri is located in the westernmost part of Assam, India, bordering Bangladesh to the west and bisected by the Brahmaputra River and its tributaries. Communities here rely heavily on agriculture and fishing, with cultivation largely restricted to paddy, jute, and mustard, and with limited exposure to improved farming techniques. Access to basic infrastructure and essential services is minimal, and annual floods regularly cause displacement and disrupt livelihoods (Deka and Kalita 2024).

As per the 2011 Census, Dhubri had a population of ⁓1.95 million, with 89.55% residing in rural areas and a high population density of 896 persons per square kilometre (Census of India 2011). Dhubri also has one of the lowest literacy rates in Assam, recorded at 58.34%, significantly below the national average of 74.04% (Census of India 2011). The district exhibits high levels of multidimensional poverty, particularly in the Char areas, which are among the most deprived regions in the country (Nath et al. 2021, Deka and Kalita 2024). According to the National Family Health Survey, 48.5% of children under 5 years old are stunted, 38% are underweight, and 73% are anaemic, making Dhubri one of India’s worst-performing districts on child nutrition indicators (NFHS-5 2021).

In 2018, Dhubri was designated an aspirational district under the Government of India’s Aspirational Districts Programme, a flagship initiative aimed at accelerating progress in key development areas across 112 of the country’s most underdeveloped districts (NITI Aayog 2022).

### Data collection and analysis

We began with a systematic mapping of the actors and institutions involved in delivering nutrition interventions. This initial phase drew on policy documents and was supplemented by a set of preliminary key informant interviews (KIIs) with district- and block-level health officials. The mapping exercise informed the development of semi-structured interview guides tailored to diverse respondent groups, ensuring the capture of both institutional logics and lived experiences.

Subsequently, primary qualitative data were collected between February and April 2022 using in-depth interviews (IDIs), focus group discussions (FGDs), and KIIs. In total, 28 IDIs, six FGDs, and four KIIs were conducted across selected villages in Dhubri. Participants included frontline health workers such as accredited social health activists (ASHAs), anganwadi workers (AWWs), and auxiliary nurse midwives (ANMs), elected representatives, members of Aanganwadi Management Committees (AMCs) and Village Health Sanitation and Nutrition Committees (VHSNCs), and direct beneficiaries such as pregnant women and mothers of children under 5 years. Supplementary Table S1 summarizes the distribution of IDIs, KIIs, and FGDs, see online supplementary material.

Interviews and FGDs employed primarily open-ended questions, probing into the day-to-day activities of frontline workers, operational dynamics of village-level committees, institutional functions, and community access to and perceptions of nutrition services. Discussions also explored challenges and enablers in implementing nutrition interventions within the specific socio-environmental context of Dhubri. All interactions were conducted in Assamese and Bengali, audio-recorded, translated verbatim, and transcribed into English.

Data were analysed using conventional content analysis, allowing codes and themes to emerge inductively from the data without pre-set coding frameworks. Initially, ⁓60 clusters of meaning were identified. They were subsequently grouped into 19 specific themes and then consolidated into 9 overarching thematic categories. These 9 categories were grouped into composite themes that are discussed in this paper.

To make sense of the thematic interconnections and relational dynamics emerging from the data, code mapping was used during the analytical phase. The resultant visual, presented as supplementary Figure S1, see online supplementary material, helps with tracing and reflecting on the complex interlinkages between various levels of influence that shape the functioning of nutrition interventions in Dhubri.

## Results

### Introduction to the actors and institutions at the village level

At the core of the formal health system for the implementation of evidence-based nutrition intervention at the village level in Dhubri are three key frontline actors: ASHAs, AWWs, and ANMs. These workers serve as the primary link between the health system and beneficiaries, particularly pregnant women and young children. Their work spans service delivery, data reporting, health education, and mobilization, and they often operate beyond their designated remits due to chronic understaffing and fragmented directives (Bhatia 2014, Closser and Shekhawat 2022).

Frontline workers operate within and alongside several community-level institutional bodies. Two such bodies—Anganwadi Level Monitoring and Support Committees (ALMSCs) and VHSNCs—-serve as participatory governance mechanisms. ALMSCs typically include AWWs, elected representatives (ER), peer mothers, and community leaders such as teachers or retired officials. They are tasked with monitoring service delivery and mobilizing local support. VHSNCs, which draw members from ALMSCs, Panchayat leadership, and local non-governmental organizations (NGOs), are envisaged as broader planning and coordination platforms under the NHM. Their effectiveness, however, varies significantly across sites depending on capacity, participation, and local power dynamics (Scott et al. 2017, Dhiman et al. 2020).

**Figure 1:**
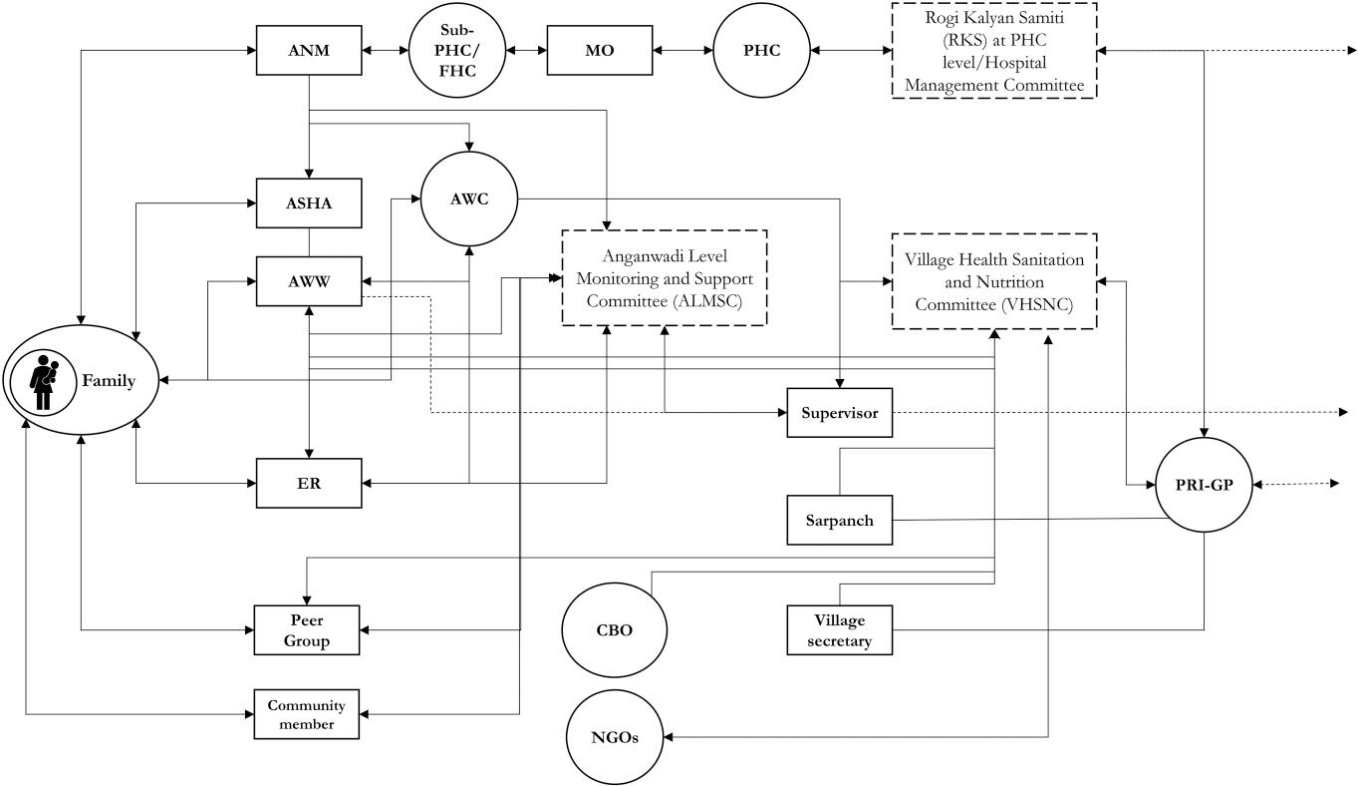
Actors and governance mechanisms in village-level nutrition service delivery in Dhubri.

Figure 1 illustrates the formal structure of this village-level nutrition intervention system, highlighting the interconnected roles of frontline workers, monitoring committees, and elected representatives. However, the study data reveal that this schematic view only partially captures the real-world complexities. While development partners and NGOs are listed as members of ALMCs and VHSNCs, their involvement in the study villages was largely symbolic. Their operations were primarily coordinated through the Child Development Project Officer (CDPO) offices, and their village-level presence was not observed during the study period. Respondents reported that even agencies responsible for distributing take-home rations brought supplies only up to the CDPO or field offices, from where frontline workers had to collect them. On the other hand, informal actors and informal practices play a critical role in sustaining the system. Elected ward-level representatives also wield considerable influence, both in mobilizing resources and shaping perceptions of accountability, even as their formal responsibilities remain limited.

### Themes from qualitative data analysis

#### Waxing and waning with the river

The Brahmaputra River dictates the rhythms of village-level health systems in Dhubri, influencing the delivery, sustainability, and effectiveness of nutrition interventions.

During the monsoon season (June to September), intense flooding and shifting river courses cause significant disruptions in the provision of essential health and nutrition services. Respondents highlighted that during floods, many communities become temporarily isolated, hindering access to critical services such as immunizations, maternal health check-ups, and nutritional supplies. During floods, anganwadi centres (AWCs), serving as the primary healthcare facilities, are frequently rendered inaccessible, damaged, or even destroyed by the floodwaters.

An elected representative described the vulnerability of the local health infrastructure:

‘This year’s flood has completely destroyed the Anganwadi Centre (AWC), disrupting services and undermining community trust.’ (Elected representative, IDI-3).

An ICDS supervisor emphasised the scale of the impact:

‘Out of 76 centres, most are damaged by floods every year. The floor is ruined, and there are big holes. Kids run around and hurt their legs. Mothers have stopped sending their children. This year, only six centres have been repaired despite repeated complaints.’ (ICDS Supervisor, IDI-25)

Community trust is undermined due to the repeated disruptions and prolonged delays in repairing damaged infrastructure, which convey a sense of neglect and inefficiency from health-system authorities. When essential services such as regular immunization schedules and nutrition monitoring are frequently interrupted, community members become sceptical about the reliability and accountability of local health institutions. Such erosion of trust negatively impacts community engagement and willingness to actively participate in health initiatives.

Frontline health workers recounted their heightened struggles during these periods, detailing logistical barriers such as damaged roads, inaccessible routes, and the need to use multiple modes of transportation. One health worker explained the complex logistics involved:

‘To bring the materials to the centre (AWC), first I have to take a bike to the ghat, take a boat, and then take a rickshaw, and then again a boat to reach the office. Only a small amount is provided for transportation—around 80 rupees—but I end up spending at least 600 rupees.’ (AWW, IDI-17)

This expenditure is nearly eight times the official reimbursement and represents a significant financial burden. The time and effort involved often eat into hours meant for rest or caregiving at home, underscoring how systemic neglect disproportionately shifts the economic and emotional cost onto the shoulders of frontline health workers.

Moreover, the floods significantly alter the region’s geography, causing households and entire communities to relocate frequently. This reshaping of local geography increases the time and effort required for frontline workers to reach each household. Despite these substantial changes, the number of houses frontline workers are expected to cover remains unchanged, creating unrealistic workloads and expectations.

The disruptions extend beyond immediate service delivery; they reverse nutritional gains made during the dry months, exacerbating issues such as malnutrition, food insecurity, and poor health outcomes among children and mothers.

Conversely, the dry season brings its own set of unique environmental pressures that further complicate service delivery. Respondents reported severe disruptions due to strong winds, which frequently damage the roofs of AWCs, leaving the facilities unusable and vulnerable to weather conditions. One AWW illustrated this scenario:

‘During the dry season, strong winds uproot roofs of AWCs. Last year, our roof blew away into the paddy fields. Although we submitted reports, no help arrived promptly.’ (AWW, IDI-6)

Maintaining the continuous delivery of health services under these changing circumstances would require additional resources and strategic adaptations that are currently lacking in policy frameworks. Respondents consistently highlighted a substantial gap between existing policy frameworks and the specific environmental and social realities of flood-prone regions. Current policies are largely predicated on stable environmental conditions, without the necessary flexibility and adaptive strategies for flood-prone areas. The absence of specific strategies leaves communities continuously vulnerable, forcing frontline workers and community members to rely heavily on precarious and often unsustainable informal coping mechanisms. Also, it is to be noted that no active involvement of Non-governmental Organisations (NGOs), International Non-governmental Organisations (INGOs), or development partners was reported in AWC reconstruction or in strengthening resilience infrastructure. While some of these organizations are formally associated with district- or block-level ICDS offices, their absence at the village level was striking, particularly given the emphasis on ‘last-mile’ delivery in donor discourse.

#### Creation of frontline families

Frontline health workers in Dhubri are responsible for delivering a wide array of services under multiple schemes and departments. This includes maternal and child health care, nutrition surveillance, immunizations, family planning, health education, disease prevention, and real-time digital reporting. The workload is compounded by overlapping responsibilities, frequent directives from multiple authorities, inadequate staffing, and the lack of clear task demarcation. One AWW captured the intensity of these demands:

‘Are we machines? We get tasks from four different departments at once. Before finishing one thing, another instruction arrives. It’s endless.’ (AWW, IDI-22)

Many workers described feeling overburdened, citing excessive travel, physical exhaustion, and emotional strain. In flood-prone and geographically isolated settings, these challenges are further intensified by the time, effort, and personal costs required to reach beneficiaries. ICDS supervisors, too, face constraints in managing large numbers of centres with inadequate staff.

The extensive demands placed on frontline health workers have necessitated family involvement in service delivery, significantly blurring the boundaries between their professional and personal lives. Anganwadi workers and ASHAs frequently described situations where their spouses, children, or extended family members became active participants in their professional responsibilities, often due to resource constraints and logistical challenges. Family members helped with tasks such as transportation of supplies, managing communications, and even participating in community outreach activities. One AWW remarked,

‘My husband goes and collects the goods most of the time… they call him only; he knows more than I do.’ (AWW, IDI-10)

This phenomenon has effectively extended the frontline workforce into what this paper refers to as ‘frontline families’. Communities frequently view these ‘frontline families’ as de facto representatives of the health system, increasing pressure on family members to deliver services effectively. This perception further complicates professional and personal dynamics, often placing workers and their families in difficult positions where they must mediate between community expectations and systemic limitations.

This extended involvement also generates significant emotional, financial, and physical strain on family members. Workers described how their familial obligations and professional duties intersected, creating tension and stress within households. Delays in salary payments exacerbated financial pressures, leading to frustration and resentment among family members. As one frontline worker articulated,

‘I have not received my salary for months. My husband complains because we are spending more on my job than we get back.’ (AWW, IDI-27)

In one of the instances, a frontline worker lamented

‘. . . my son, daughter, everyone in the family is involved actually. I keep asking them for some kind of help. Sometimes they say no, but I have to keep asking, there is no other way.’ (ASHA, IDI-1)

Many frontline workers like her expressed deep personal conflict regarding the involvement of their children in their work, particularly when this participation led to neglect of their own children’s needs. As another AWW elaborated on this,

‘I have only one daughter; my husband passed away three years ago. I go to work, leaving my daughter at home. She often has to eat cold food and sometimes gets emotional. As no one else is at home, I lock her inside, scared for her safety.’ (AWW, IDI-15)

In some cases, workers resorted to measures such as sending their children to hostels to ensure their well-being while fulfilling work obligations.

‘I had to send her to a hostel. It is not easy to keep her with me as it affects my work, but I have to fulfil my duties. My daughter understands that I work for social welfare and is proud of me, but she is malnourished and cries a lot’ (AWW, IDI-2).

Such decisions highlight the stark personal sacrifices workers make due to systemic shortcomings.

#### Double-edged sword of community participation

Community participation is often promoted as a cornerstone of effective health-service delivery, particularly in contexts where formal health-system reach is limited. In Dhubri, local participatory coordination structures such as Anganwadi Management Committees (AMCs) and VHSNCs provide the institutional space through which community members engage with and shape nutrition interventions. This participation takes multiple forms—monitoring services, assisting in ration distribution, facilitating outreach, and mobilizing beneficiaries for meetings and campaigns. At its best, community engagement enhances trust, leverages local knowledge, mobilizes resources, and helps tailor services to local needs.

However, this study reveals that community participation in Dhubri is deeply ambivalent and often functions as a double-edged sword. While it enables localized problem-solving and greater visibility of programmes, it also generates unrealistic expectations and adds to the burden and scrutiny faced by frontline workers.

AWWs reported that community members frequently held them responsible for infrastructure failures, service delays, or insufficient rations-issues beyond their control. For instance, some committee members expected the AWWs to act as tuition teachers, shifting the focus of AWCs to education. One worker explained,

‘The committee keeps asking why the children don’t know their letters. They want me to sit with them like a schoolteacher. But my main work is nutrition and health.’ (AWW, IDI-6)

AWCs are rural childcare centres under the ICDS programme, and most of the interventions converge at these centres at the grassroots. However, perceptions of what AWCs are meant to deliver vary widely within communities. Some view them as preschools, others as ration distribution centres, day-care facilities, or local government offices. While they do provide some pre-school education, distribute supplementary ration, and function as day-care to some extent, the priority for the Anganwadi worker is nutrition monitoring and surveillance. The plural perceptions of the AWCs, thereby, shape how community members interact with the space and with the workers, often complicating the delivery of nutrition-specific services. AWWs described the challenge of navigating these expectations, especially when community members demanded services that aligned more with their personal or collective aspirations than with the scheme’s formal mandate.

In some instances, community participation went as far as assigning informal duties to family members of frontline workers. One case involved the son of a deceased Anganwadi helper (each AWC is staffed by an AWW and an Anganwadi helper, who supports the AWW by assisting with cooking, cleaning, and mobilizing children and women from the community) who continued to assist in running the centre without any formal payment. As described by an elected representative,

‘It is the right thing to do, right? I only suggested he help with the work until we get another helper. There is so much work, so the AWW cannot do everything alone. It is his duty to his mother and the community.’ (ALMC Member, FGD-1)

Furthermore, participation is shaped by social hierarchies and inequities. The voices most often heard in meetings and decision-making processes tend to come from relatively better-off households—those who have the time and flexibility to attend. Field labourers and poorer women are often absent from these forums, not due to a lack of interest but because participation competes with their subsistence activities. As one mother explained,

‘They call us for meetings, but I can’t go. I work in the fields from early morning till evening. The others go because they don’t have to work like we do.’ (Mother, FGD-4)

Frontline workers also reported instances where participation led to coercive dynamics. Community members sometimes demanded that AWWs contribute personal resources or even use their own property for setting up AWCs. In one case, an AWW was pressured to construct a centre on her own land due to the lack of available infrastructure. Moreover, participation occasionally turned into overreach, with AMC members disseminating inaccurate health information during awareness sessions, further complicating frontline workers’ efforts. For instance, an AWW described an incident where an AMC member shared incorrect dietary advice:

‘. . he said pregnant women should not eat eggs. The supervisor was present at the event, and she clarified it was a misconception, but without her, I couldn’t have corrected him’ (AWW, IDI-2)

Despite these challenges, many workers recognised the potential value of community participation, particularly when it translated into support for resource mobilization or resolving immediate infrastructural problems. Strengthening community participation requires investment in training, clearer role definitions, and institutional safeguards that protect frontline workers from being unduly burdened or scapegoated. Without such efforts, participation may continue to be both a tool for strengthening interventions and a pathway for diffusing accountability downward in already fragile systems.

#### Informal spaces as the engine

Amid the constraints of limited formal infrastructure, fragmented coordination, and unpredictable policy support, it is often informal spaces that keep the machinery of frontline health delivery moving in Dhubri. These spaces—both physical and relational—serve as essential engines for the functioning of village-level health systems.

The office of the CDPO emerged in interviews as one such key space. Supervisors and frontline workers gather there not just for official business but for informal interactions that help troubleshoot problems, share field experiences, and develop joint strategies. A supervisor shared,

‘Two days a week, we sit in the office. There, we get to know about stories from the field, jokes, and all. This is helpful for us, actually; it helps with the work and improves our experience. We decide on things and do planning also together.’ (ICDS Supervisor, IDI-18)

These interactions are often spontaneous, yet they allow for real-time adaptations, peer learning, and mutual support that formal systems are too rigid or too fragmented across schemes and reporting lines to enable.

Similarly, village health sanitation and nutrition days, which are officially intended for service delivery and beneficiary outreach, also serve as critical informal coordination hubs. During village health sanitation and nutrition days, AWWs, ASHAs, and ANMs often use the occasion to share information, align their activities, and discuss local challenges.

Digital platforms, particularly WhatsApp groups, have become vital informal communication tools. ASHAs and AWWs use these groups to exchange updates, coordinate logistics, resolve doubts, and share motivational messages. One AWW noted,

‘We have WhatsApp groups where we check on each other, ask questions, and give reminders about duties. If someone has a problem, others jump in to help.’ (AWW, IDI-15)

These networks, while unofficial, function as a support system that reduces professional isolation and bridges gaps in formal communication channels.

Informal arrangements also extend to sharing physical resources. Weighing machines, mobile phones, and educational materials are borrowed or rotated between centres when equipment is lacking or non-functional.

The strength of these informal spaces lies in their flexibility and responsiveness. They facilitate bottom-up problem-solving and foster camaraderie among overburdened workers. There is a tacit understanding among frontline workers that they must rely on each other to fill systemic voids.

In Dhubri, these informal spaces are not just supplementary—they are indispensable engines that keep the system moving. However, despite the positive contributions, these arrangements of informal spaces are often invisible to higher levels of administration and remain unsupported and unsustained by policy. Recognising and investing in these informal spaces could transform them from stopgap solutions into integral components of resilient health systems.

#### Campaigns without context

Campaigns such as National Nutrition Month (Poshan Maah) and special nutrition drives are intended to create awareness, promote healthy behaviours, and intensify community engagement around nutrition. However, in Dhubri, these campaigns often operate in isolation from the lived realities of the communities they target. Rather than fostering sustainable behavioural change or addressing structural constraints, they frequently emphasise one-off events and symbolic gestures, creating a disconnect between messaging and practice. Most of the campaign materials, such as IEC leaflets, slogans, and visual posters, are centrally designed at the state or national level, often drawing from donor-supported communication templates. These templates often fail to account for local ecologies and socio-economic capacities.

Nutrition messages promoted during campaigns often focus on aspirational food items like apples, grapes, and dry fruits—items that are prohibitively expensive for many families. One AWW shared,

‘We give everything—apple, dates, grapes, eggs. Yes, papaya also—but some things are more nutritious.’ (AWW, IDI-6)

While these foods were distributed during special days or events, they created unattainable standards. Beneficiaries, in turn, expressed frustration:

‘They show us fruits and dry fruits in the meetings and say this is what children should eat. But who can buy all that every day? It feels like they are showing us things we cannot have.’ (Mother, FGD-3)

This disconnect fosters disillusionment. Campaigns are often accompanied by temporary resource influxes that are not sustained throughout the year. As a result, community members begin to view such efforts as superficial rather than impactful. A mother remarked,

‘What we get is sattu (ground roasted Bengal gram) and rice a few times a year from the centre, but at the meetings they advise us to eat all good food and feed the children good food. It is not possible. Things are very expensive.’ (Mother, FGD-5)

Moreover, the campaigns often fail to reflect local food practices or promote affordable, accessible, and culturally appropriate alternatives. Little attention is paid to seasonal, locally available, and cost-effective foods, such as green leafy vegetables, pulses, and regionally grown fruits. These campaigns are not only disconnected but also frequently perceived as insensitive. By showcasing food items and lifestyles that are economically unattainable for many families, they inadvertently reinforce feelings of exclusion, inadequacy, and shame. This emotional disconnect was evident in interviews with beneficiaries who described feeling judged or overwhelmed by the nutritional ideals presented during campaign events. One mother noted,

‘They tell us to give the child almonds and then look at us as if we’re not doing our job as mothers when we can’t afford them.’ (Mother, FGD-4)

Hygiene and sanitation are another set of campaign themes that illustrate this disconnect. One AWW described her approach:

‘I have explained to them many times, instructing them to ensure their children look presentable and clean before sending them to the centre. I have even contacted mothers directly to explain… if the mother is not nice, then how will the children be?’(AWW, IDI-10)

Campaign messaging frequently positions cleanliness as an individual moral responsibility rather than a collective goal requiring infrastructure, support, and empathy. This framing was echoed by a community member who said,

‘They keep saying our children fall sick because we don’t bathe them properly. They act like all our problems come from being dirty. But we are farmers and labourers—of course, there will be some mud or dust on us.’ (ALMC Member, FGD-6)

Over time, these repeated disconnects, emotional disillusionment, and perceived insensitivities contribute to a growing resistance from community members towards health and nutrition interventions.

When advice is perceived as judgmental or out of touch, and when participation does not lead to tangible improvements, people begin to disengage or push back, undermining the very goals of these campaigns.

## Discussion

This study revealed five interconnected themes that shape the everyday functioning of nutrition interventions in Dhubri, a district marked by ecological precarity and systemic neglect. First, seasonal disruptions caused by the Brahmaputra River continually destabilize service continuity, exposing how national schemes premised on stable infrastructure fail to accommodate environmental volatility. Second, the emergence of ‘frontline families’ illustrates how the personal lives and unpaid labour of health workers’ households become integral to sustaining service delivery, often at considerable emotional and financial cost. Third, community participation operates as a double-edged process: it enables some local problem-solving but also reinforces hierarchies, diffuses accountability downward, and sometimes co-opts household members into unpaid roles. Fourth, informal spaces and interpersonal networks function as the true operational infrastructure of the system, bridging gaps left by fragmented formal mechanisms. Finally, centrally designed campaigns, often shaped by top-down communication templates, remain disconnected from local realities by promoting aspirational ideals and individual moral responsibility rather than collective, context-sensitive action.

### Local health system as a social system

This study contributes to the growing body of scholarship in health policy and systems research that foregrounds the complex, context-sensitive nature of health-system functioning, particularly at the interface between policy design and frontline implementation. Our findings affirm the argument that health systems are ‘social systems’ (Gilson et al. 2011, Sheikh et al. 2011) that cannot be fully understood through their formal blueprints alone. We uncover how health systems are not merely assemblages of formal actors and institutions, but dynamic, socially embedded constructs continually reshaped by environmental, political, and relational forces.

In Dhubri, the formal architecture of the ICDS and NHM schemes is animated by informal networks and community practices. Health interventions wax and wane with the Brahmaputra’s seasonal rhythms, revealing how ecological disruptions profoundly mediate the reach, continuity, and credibility of services. These findings resonate with previous work on health-systems resilience and adaptive governance in fragile settings (George et al. 2018, Gilson et al. 2020, Truppa et al. 2024), but extend this scholarship by offering a grounded account of how natural ecologies can act as core determinants of intervention trajectories.

### Invisible labour and the emergence of frontline families

A key conceptual contribution of this paper is the articulation of the ‘frontline family’ as an emergent category of health-system functionality. While existing literature has documented the burdens of task shifting and human resource constraints in primary healthcare (Lehmann et al. 2009, Keeling and Maradiaga 2022), our study reveals how these burdens overflow into the domestic sphere, with family members of frontline workers becoming de facto extensions of the state. This entanglement of household and health-system labour surfaces critical ethical and practical concerns. It disrupts the tidy boundaries between paid and unpaid work and problematizes the notion of ‘voluntarism’ that often legitimizes the use of female community health workers (Jenkins 2009, Hanlon et al. 2011). It also illustrates a broader pattern in which the state systematically extracts women’s unpaid or underpaid labour to implement its policies. Scholars describe this phenomenon as ‘care extractivism’—the global commodification of social reproduction, where female healthcare workers sustain the health system without recognition or fair compensation for their contributions (Wichterich 2020).

In doing so, it draws attention to the hidden emotional, financial, and temporal subsidies that households provide to prop up chronically underfunded systems. The emotional toll on workers and their kin further underscores the need to reconceptualize care work within public health, not merely as a professional responsibility but as a deeply relational, often feminized, and systematically undervalued form of labour (Harman 2018, 2020, Harcourt and Bauhardt 2018).

### Realities of local participation structures

Community participation is revealed here as a deeply ambivalent practice. On one hand, it provides legitimacy, local insight, and operational support. On the other hand, it amplifies role confusion, reinforces unequal power relations, and facilitates the downward diffusion of accountability. Our findings echo similar critiques from participatory governance literature, which cautions against over-romanticizing local institutions without attending to their internal hierarchies and exclusions (Cornwall 2008, George et al. 2015). Importantly, the varying expectations communities place on AWCs and frontline workers not only create communication gaps but also the institutional failure to negotiate a shared meaning of care and service.

These contested perceptions are a crucial yet under-addressed aspect of health-systems functioning that affects how interventions are experienced and enacted at the local level. The uncritical promotion of participation without commensurate support, role clarity, or attention to local dynamics often reduces it to what Campbell and Scott (2011) describe as a ‘hollow strategy’, where engagement is symbolically invoked but unevenly practised. In Dhubri, this phenomenon intersects with what critics of participatory governance have termed the ‘tyrannies’ of participation, where well-intentioned initiatives fail to deliver on their transformative promise due to entrenched power imbalances and a neglect of lived realities (Richardson et al. 2019). This convergence ultimately hinders meaningful participation and reinforces existing hierarchies and exclusions.

These patterns also reveal clear accountability asymmetries within the system. Frontline workers, being the most visible actors, are held directly accountable for service failures and expectation mismatches, while higher-level programme designers and development partners often remain beyond public or institutional scrutiny within participatory governance spaces. This uneven distribution of accountability reflects what Gaventa’s (2005) ‘Power Cube’ conceptualizes as the operation of visible and invisible power across invited and claimed spaces of participation.

### Informal spaces and networks as systemic infrastructure

Emerging research underscores how informal infrastructures can provide critical health and social support during institutional failure (Davis 2018, Onditi et al. 2021). Our study shows that informal spaces function as critical ‘shadow’ substructures of system operation (Leck and Roberts 2015). These are not peripheral but central to maintaining the fragile equilibrium of service delivery. The reliance on such informal infrastructures, however, also reflects a deep systemic precarity. Without formal recognition, these practices remain unsupported, rendering the system vulnerable to individual burnout and collective collapse. There is an urgent need to move beyond the dichotomy of formal–informal and to conceptualize informality as a modality of governance, one that can be institutionally scaffolded.

The informal relations that sustain service delivery in Dhubri can be situated within broader co-production theories (Ostrom 1996, Bovaird 2007) of public service delivery. This study demonstrates how such co-production extends beyond formal participatory structures to encompass domestic, kinship, and neighbourhood networks that routinely substitute for absent institutional support. In doing so, this study adds to the literature by drawing attention to the intimate and invisible forms of co-production that underpin public health delivery in contexts of chronic under-investment, and by reframing them as both a source of resilience and a manifestation of structural inequity.

### The disconnect of symbolic campaigns

Nutrition campaigns like Poshan Maah emerge in our study as emblematic of a broader disconnect between state aspiration and local capability. They reflect what Gupta (2012) might describe as the ‘symbolic state,’ where policy visibility is prioritized over substantive engagement. While intended to promote awareness and behavioural change, these campaigns often misalign with local socioeconomic realities, leading to feelings of shame and alienation rather than empowerment. This misalignment is not simply a matter of poor design but points to a deeper failure to ground policy in local epistemologies and material constraints. In this light, the politics of representation and inclusivity must be placed at the heart of any community participation to avoid top-down directives that feel disconnected from everyday contexts and lived realities. Effective community participation demands redistributing power to those embedded within the health system’s grassroots architecture, recognising them not merely as implementers but as co-producers of health knowledge and practice, without which the promise of equity and responsiveness in public health shall remain largely rhetorical.

### Implications for health policy and systems thinking

The findings from Dhubri urge a rethinking of how implementation is conceptualized in health policy. It is not a linear translation of programme guidelines, but an inherently negotiated process embedded in everyday lives, shaped by rivers, relationships, histories, and hardships. Systems thinking approaches must take seriously the relational, ecological, and emotional dimensions of health-system functioning (Sheikh et al. 2011, Gilson 2012).

This study also challenges the adequacy of metrics-focused assessments of intervention performance. Traditional monitoring frameworks may fail to capture the informal labour, coping mechanisms, and contextual negotiations that underpin delivery. As calls for data-driven policymaking grow louder, it is imperative to complement quantitative indicators with qualitative insights that capture the system’s ‘invisible work’ (Scott et al. 2019). Recognising the invisible work requires human-resource policies that formally acknowledge and compensate unpaid support, thereby reducing reliance on invisible care economies. Policy frameworks must move beyond uniform implementation models to incorporate adaptive planning for ecologically fragile and politically marginalized contexts. Designing flexible infrastructure budgets, maintaining contingency funds for seasonal disruptions, and institutionalizing local feedback loops could enhance both accountability and resilience.

### Strengths and limitations

This study offers several strengths. Conceptually, it advances understanding of village-level health systems by foregrounding the lived realities of frontline actors and communities in contexts marked by informality, ecological precarity, and systemic neglect. The analysis moves beyond a programmatic lens to illuminate how care, accountability, and adaptation are negotiated within everyday service delivery.

At the same time, several limitations should be acknowledged. The research did not include development partners or donor agencies in the sample, as they did not emerge as active village-level stakeholders during the initial mapping. In Dhubri, such agencies primarily interface with the CDPO office rather than with frontline workers or communities. Nevertheless, their perspectives could offer valuable insights into how external actors influence local nutrition interventions.

Similarly, while the study mapped the actors and institutions involved in service delivery, it did not undertake a stakeholder analysis in the formal sense of ranking actors by power and influence. This was a deliberate decision, as the study aimed to qualitatively examine the social functioning of the village-level health system and understand how actors and institutions collectively shape nutrition interventions. A stakeholder matrix would have shifted the analytical focus toward comparative power mapping, which lay beyond the objectives of this paper. Future research could build on this by mapping relevant actors and stakeholders and their levels of influence and interest.

While every effort was made to ensure reflexivity and linguistic accuracy through the use of local facilitators and peer debriefing, nuances may still have been shaped by translation and interpretation processes.

## Supplementary Material

czag011_Supplementary_Data

## Data Availability

The data underlying this article cannot be shared publicly for the privacy and safeguarding of individuals that participated in the study. The data will be shared on reasonable request to the corresponding author.

## Reflexivity statement

The authorship group comprises individuals from different institutional and disciplinary backgrounds, genders, and levels of seniority. The first author is a male early-career researcher with a total of 7 years of experience, including 5 years during his PhD and 2 years post-PhD. The second author is a female public health researcher with >10 years of experience, including a wide range of freelance work and research projects. The third author is a senior female academic and professor with 15 years of experience in teaching and interdisciplinary research. The authors acknowledge their positionality as external researchers to the community studied, which might influence interactions with participants and the interpretation of data. The researchers come from relatively more privileged caste and class locations than the participants, who, as described in the manuscript, reside in a politically turbulent and socioeconomically marginalized region. These differences may have shaped some of the information shared by participants, particularly around issues such as linguistic identity, religious practices, and diet. The first author, who led the fieldwork, did not speak the regional languages, while the third author possessed intermediate proficiency. All interviews were conducted with the help of trained local facilitators. Translation and transcription were performed by native speakers of Assamese and Bengali under the supervision of the third author. Efforts were made to minimize power imbalances during data collection by employing culturally sensitive methodologies. Continuous reflection and peer-debriefing were integral components throughout the research process to maintain credibility and ethical standards.
