# Supplementary material for: A river, frontline families, and the double-edged sword of community participation: how nutrition interventions are shaped at the village level in Dhubri, India
Source: Health Policy Plan. 2026 Jun 29;41(Suppl 1):i71–82. doi: 10.1093/heapol/czag011 (PMC13311669; doi:10.1093/heapol/czag011)

*Appendix Table 1: Distribution of data collection methods.*

| **No** | **ICDS Block** | **Village** | **IDIs** | | | | | **KIIs** | **FGDs** |
| --- | --- | --- | --- | --- | --- | --- | --- | --- | --- |
|  |  |  | ASHA | AWW | ANM | ER | ICDS Supervisor | CDPO |  |
| 1 | Block 1 | *Village 1* | x | x |  | x |  | x |  |
|  |  | *Village 2* |  |  |  |  | x |  | ALMC Members |
| 2 | Block 2 | *Village 3* | x | x |  |  |  | x |  |
|  |  | *Village 4* |  |  |  |  | x |  | VHSNC Members |
|  |  | *Village 5* |  |  |  |  | x |  |  |
| 3 | Block 3 | *Village 6* | x |  |  |  |  |  |  |
|  |  | *Village 7* |  | x | x | x |  |  | Mothers Group |
| 4 | Block 4 | *Village 8* | x | x | x | x |  |  |  |
| 5 | Block 5 | *Village 9* |  | x |  |  |  |  | Mothers Group |
|  |  | *Village 10* | x |  | x |  | x |  |  |
| 6 | Block 6 | *Village 11* | x |  |  |  | x | x |  |
|  |  | *Village 12* |  | x |  |  |  |  |  |
|  |  | *Village 13* |  |  |  | x |  |  | Mothers Group |
| 7 | Block 7 | *Village 14* | x | x | x |  | x | x |  |
|  |  | *Village 15* |  |  |  |  |  |  | ALMC Members |
|  |  | *Total* | 7 | 7 | 4 | 4 | 6 | 4 | 6 |

*Appendix Figure 1. Interlinkages of themes emerging from qualitative data analysis – Code map used to visualise relationships among themes shaping nutrition intervention delivery in Dhubri.*


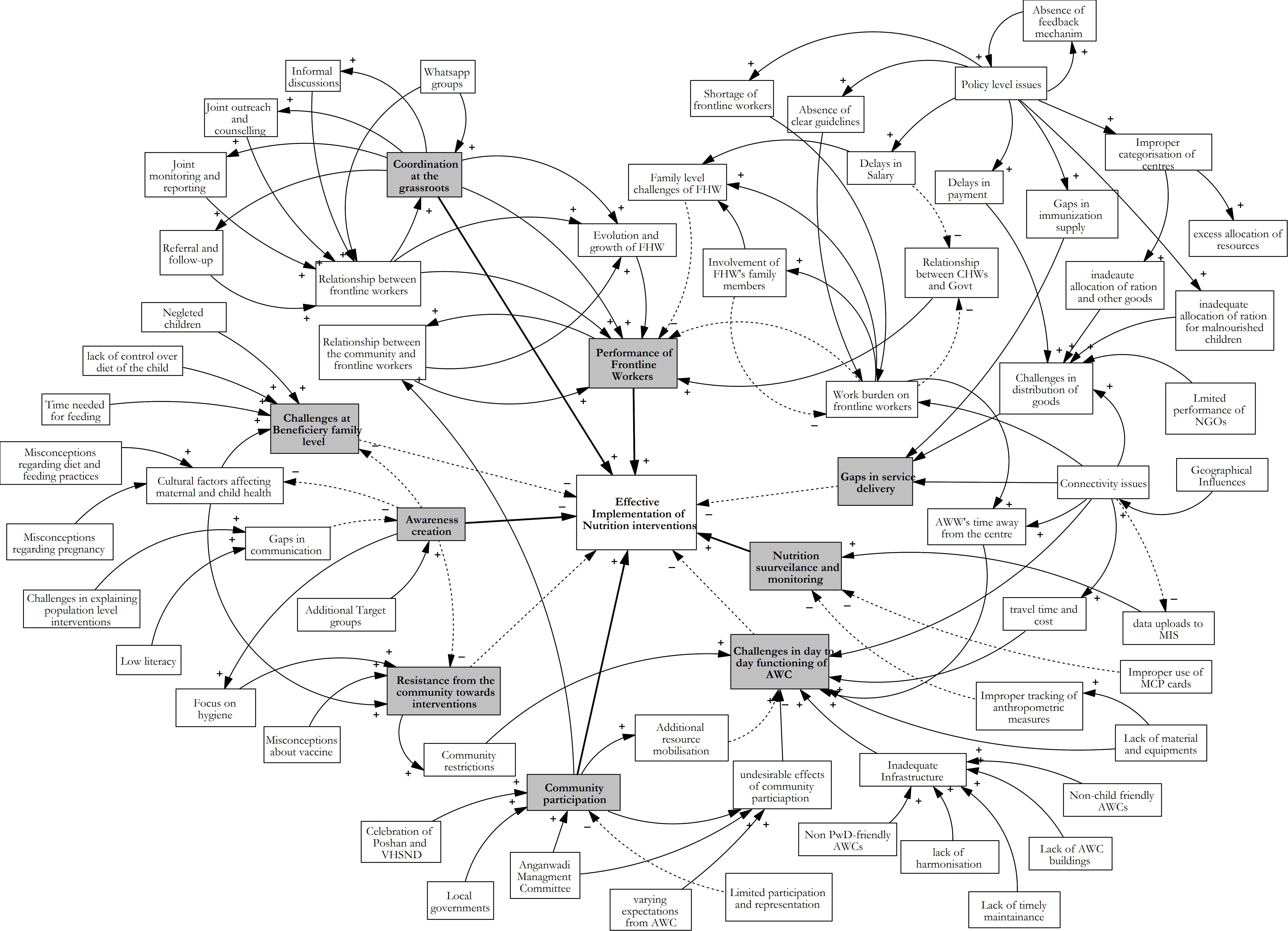

Supplement: czag011_Supplementary_Data [file czag011_supplementary_data.zip › Supplementary Files.docx]
