# Supplementary figures and images for: A river, frontline families, and the double-edged sword of community participation: how nutrition interventions are shaped at the village level in Dhubri, India
Source: Health Policy Plan. 2026 Jun 29;41(Suppl 1):i71–82. doi: 10.1093/heapol/czag011 (PMC13311669; doi:10.1093/heapol/czag011)

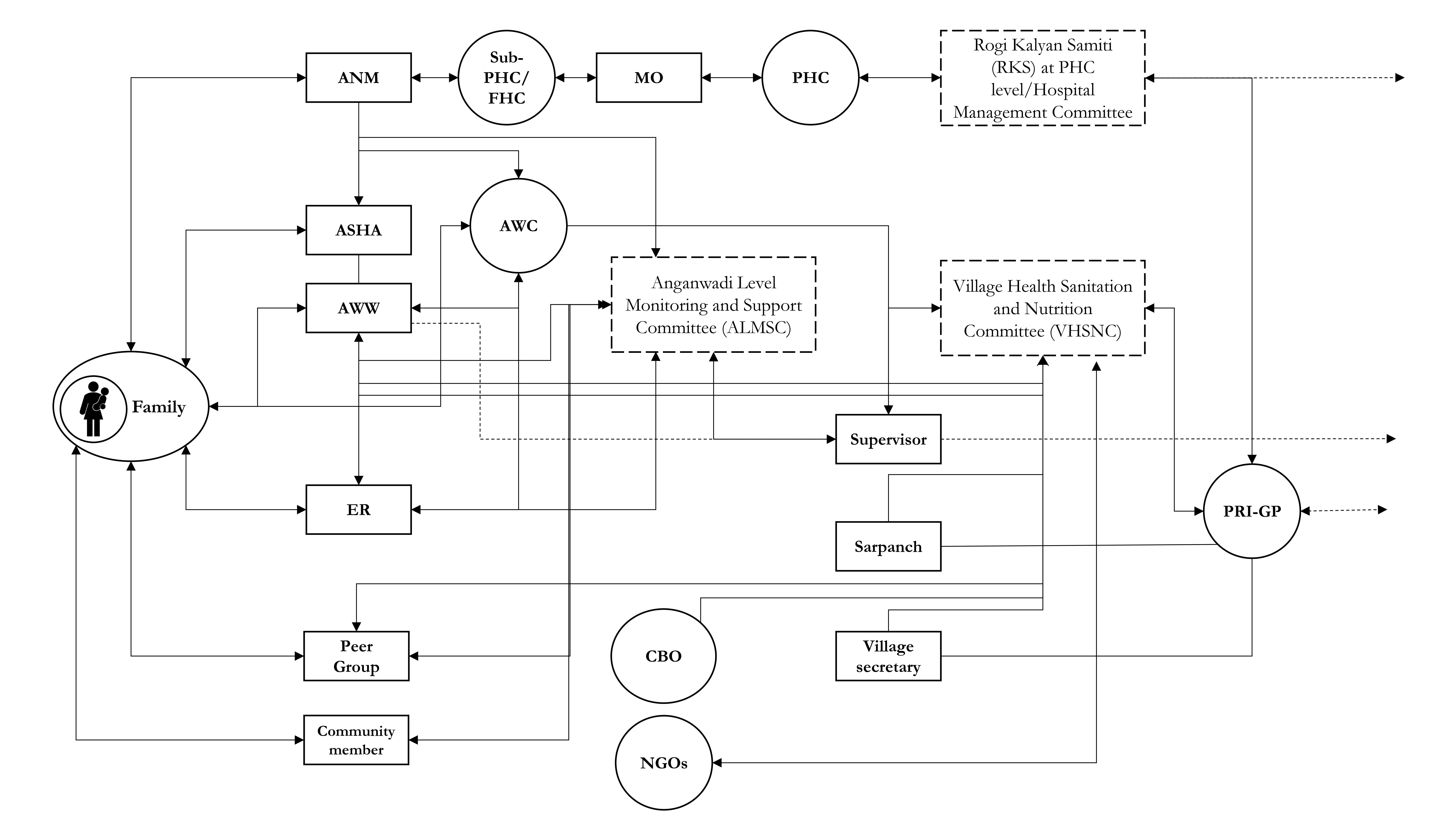

Supplement: czag011_Supplementary_Data [file czag011_supplementary_data.zip › Figure1_resized.jpg]
